# Discussion on the Diagnosis of Cancer in the French Academy of Medicine

**Published:** 1857-07

**Authors:** 


ART. II.—*Discussion on the Diagnosis of Cancer in the French Academy of Medicine.* Translated by C. B. Hutchins, M. D., Buffalo, N. Y.

The following discussion in the Academy of Medicine of Paris, is translated from a work entitled "Annuaire Des Sciences Médicales," "Par M. le Docteur Lorain," published in Paris in 1856. It is a resumé of that discussion which took place in the Academy of Medicine, and which caused such an excitement in the medical world of Europe, in 1854 and 1855.

Since the questions examined in this discussion (viz., the utility of the microscope in diagnosis and the curability of cancer,) are of great interest, I have thought it worthy of translation for your Journal.

On the 26th of September, 1854, M. Jobert read a report of a case of cancer of the testicle, in a child at the breast, addressed to the Academy by M. Pamard, correspondent at Avignon. At the end of the report a discussion sprang up between MM. Robert and Velpeau, on the curability of cancer, and this question, at the request of M. Velpeau, was made the order of the day for the next sitting: such was the origin of the discussion upon cancer.

### *Sitting of October* 3, 1854.

MM. Robert and Velpeau not taking the floor, M. Leblanc spoke on the question. He recognized several kinds of cancer: true cancer, epithelial cancer, melanic cancer, etc. The first variety reäppears very often after an operation: the epithelial less often, and the melanic oftener than either of the others—even than the true cancer. The varieties of cancer are not always pure; the tissues of different kinds are often found in the same case:

then the disease is the more serious according as there is more of the true or melanic variety present. The observations of M. Leblanc extended to animals as well as to men.

M. Barth spoke in his turn and asked whether the microscope was useful in diagnosis, a question which he decides in the affirmative; but yet the microscope is not indispensable. The use of the microscope ought not to cause the neglect of anatomy, studied with the naked eye; the scalpel shows that the cancer type has a solid woof, a substance in which is infiltered a special pulp. This pulp appears to M. Barth to have great importance: it seems to him easy to understand that the cancer cell is not found in all of the different stages of cancer.

As to the curability of cancer, M. Barth believes that cancer is rarely curable—more rarely than phthisis. The fear of the diathesis should not hinder a surgeon from removing a cancerous tumor. It is better to change the conditions in which a cure might follow than to preach incurability. Of these conditions a few are known. Cancer is the more grave when softened, ulcerated and the lymphatic ganglia engorged. There are more chances for cure if the cancer is solitary.

### *Sitting of October* 10, 1854.

M. Robert brings to mind that this discussion arose from an opinion expressed by him, that the cure of cancer, especially of the encephaloid variety, is of rare occurrence. It appears that M. Velpeau is not of this opinion. M. Robert is astonished at the enormous proportions of cures announced by M. Velpeau, in his recent treatise on maladies of the breast. He brings to mind that M. Broca has given the result of nineteen tumours operated on by Blandin, and whose cancerous nature had been verified by a microscopic examination. None of these nineteen cases had been exempt from relapse in a space of time not going beyond two years. M. Lebert, in his treatise on cancerous diseases, says, that of thirty-four cases operated on, six died in the operation; twenty-one had a return of the disease in a lapse of time varying from three months to two years; and seven were lost sight of. Were those veritable cancers which M. Velpeau had cured so many times? Were there not in this number some benign tumors? M. Velpeau has not always known so well as now, how to distinguish tumors of the adenoid species. Besides, if one refers to the study of tumors in general, in different organs, frequent causes of error in diagnosis are found. The microscope has shown that independently of hypertrophic tumors, there are epithelial and fibroplastic tumors; and observation has shown that these last productions return much less frequently than cancer. It is necessary to put an end to the confusion which to-day reigns in the question of cancer.

M. Velpeau, in answering M. Robert, replies first to the argument which consists in throwing suspicion on the validity of his diagnosis. Mr. Robert is astonished at the number of cures I have given. I have less than any other the pretension to infallibility. But I can testify, without the fear of contradiction, that in a great number of cases the cancer presented itself with characters so distinct, so evident, that error was impossible, or if it happened, so rare as not to be worthy of mention. M. Lebert has never given a single denial to my diagnosis distinctly given in these evident cases.

M. Velpeau agrees that there are benign tumors which are so similar to those of a malignant character, that it is difficult to decide to which variety they belong.

It is on account of these difficulties of diagnosis that, wishing to call to his aid every means of reaching the truth, M. Velpeau had encouraged the labors of the microscopists, and he has now come to distinguish cancers from benign tumors, called hypertrophic, adenoid and fibrinous. After leaving out these tumors called cancerous, about one-fourth of those which had before been confounded together, M. Velpeau wished to establish classes among cancers, with regard to their tendency to return, and he had been able to distinguish cancers, which, removed by an operation, nearly always return: those which return often, those which return sometimes, and finally, those which return but rarely.

M. Velpeau recounted briefly the history of a lady on whom he operated in 1836, for an ulcerated encephaloid cancer of the breast, and who was entirely cured, and remained so until the present time. M. Velpeau cited two other analagous observations. To escape the reproach of incertitude in diagnosis, M. Velpeau says that since 1847 he has caused all the tumors which he has removed to be examined by the microscopists. As to the possibility of a return he thinks that sufficient time has elapsed since the operation, (seven or eight years) to put them beyond fear of the like.

M. Robert accuses M. Velpeau of confounding epithelial tumors with cancer. I regret, says M. Velpeau, that this question of epithelial and fibro-plastic tumors, obliges me to throw a stone into the camp of the microscopists. Among the heteromorphous (heterologous of Laennec) tumors, the microscopists place schirrhus and encephaloid, or to speak otherwise, what by everybody else is considered the genuine type of cancer; among the homœomorphous are found epithelial and fibro-plastic tumors; but in the same class are placed, without offering any different microscopic character, warts, corns, venereal condylomes, etc., etc. Now I should like to know if in a clinic it will be possible to class these frightful ulcers, which destroy first the lips, then all the face, with warts and corns?

M. Velpeau recounted how M. Lebert had at first the illusion that the homœomorphic character excluded a reäppearance, or admitted it very rarely. But experience taught the contrary, and it was henceforth recognized by M. Lebert and his pupils, that not only epithelial tumors could reproduce themselves, but that they could attack the neighboring ganglion in the manner of veritable cancer. That is not all: a cancroid of the lips is removed and a little later the cancerous tissue develops itself, as was proved microscopically, in the superior maxillary, far from the original point of disease.

Moreover, these cancers kill as the other varieties. They may be called epithelial, if it is desirable, but they must still retain the name of cancer.

Let us pass now to tumors that the microscopists call fibro-plastic, and which constitute. according to them, another class of homœomorphic tissues. M. Velpeau says that he has always been arrested by this denomination of homœomorphic tumor, given to an affection which increases and develops itself so as certainly to cause death, unless removed in time. The indurated base of a venerial chancre, and the kaloid, are composed of fibro-plastic tissue. But how can these lesions be compared with tumors so different, which spring up everywhere, acquire promptly enormous proportions, insinuate themselves into all of the tissues, and certainly result in death. The microscopists say that fibro-plastic tumors are homœomorphous and benign; and as proof they affirm, that once removed these tumors do not return. M. Velpeau demonstrated that this proposition was false, and in support of his opinion, he cited at length several remarkable facts, where fibro-plastic tumors which had been operated on, returned and infected the whole economy. According to M. Velpeau these fibro-plastic tumors reproduce themselves in the same manner as cancers. Moreover, where is the proof that the cancer cell, which they invoke can constitute an element distinguishing between two kinds of tumors. The microscopists themselves do not agree. The disciples of M. Lebert deny the cell: for them it is the nucleus which constitutes the distinguishing characteristic of cancer. I no more believe, says M. Velveau, that the nucleus of to-day can be the essential characteristic of cancer, than I believed in the cell of yesterday. After having reported several observations where cancerous tumors were cured by an operation, and tumors, not cancerous (according to the microscopists) reäppeared and caused death, and where cancerous tumors followed a cancroid, M. Velpeau thus defines his opinions: "Such, gentlemen, are the reasons which make me say that the microscope has not served to enlighten us in the diagnosis of tumors. There still remains an ignorance of cancerous tumors that the microscope has not enlightened. Then have I not a right to ask what has been the utility of the microscope in clinics? Of what use is it in view of the incertitude in which it leaves us? What reliance can be placed on it, if it reveals to us an element which is considered as characteristic of cancer, in tumors which are not cancerous; and if by the side of that it does not find this element in tumors, which from every other character are manifestly cancers?" M. Velpeau does not proscribe the microscope, he is only opposed to the infatuation of which this instrument is the object.

*Sitting of October* 17*th*, 1854.

M. Cloquet, after having complimented the experience and skill of the speakers who had preceded him, and declared himself a partisan of the opinions sustained by M. Velpeau, said that according to his views, all of the affections grouped under the name of cancer, do not have the same tendency to return. He cited a case of Noli me tangere cured by him with arsenical paste; he hesitates to believe that this Noli me tangere belongs to the class of cancers. True cancer constitutes a terrible family, each member of which distinguishes itself, by its greater or less aptitude to reproduction.

Several divisions can be established between the different members of this family, and these divisions rest at the same time on their anatomical character and upon their degree of malignity. M. Cloquet places in the first rank the melanic cancer, the incurability of which appears to him beyond question, since he has decided never to operate on another. After the melanic cancer in order of malignity, comes the encephaloid, then the schirrhous, cartilaginous, colloid, epithelial, and finally, the affection named fibro-plastic, which must be ranked among cancers.

M. Cloquet insists on considerations of surgical order which ought to direct the practitioner; the position and development of the tumor, engorgement of the lymphatic ganglia. * * * The prognosis is often doubtful. There is no absolute certainty. * * * M. Cloquet reported several cases of cancerous tumors cured by an operation, contrary to all expectation. The question of the utility of the microscope was scarcely brought into question during the remarks of M. Cloquet, only that he thinks it cannot well be dispensed with in clinical examinations.

*Sitting of October 24.*

M. Robert, in replying principally to M. Velpeau, said that observations made twenty or thirty years since, were not entitled to the same consideration as those of later date, because diagnosis had been much improved in the last few years, a progress in which the microscope had its share. As to the cures obtained by M. Velpeau during the last few years, are they not too recent to affirm that a return of the disease will not take place; has not M. Velpeau himself, in his treatise on diseases of the breast, cited examples of return a long time after the operation?

M. Robert returns to the question of diagnosis: he does not believe in infallibility. There are difficult and doubtful cases in which M. Velpeau himself hesitates to decide absolutely: then the microscope is consulted and valuable information is obtained of the intimate nature of tumors. Such was the case in a female, operated on by M. Velpeau, for a benign tumor; but upon the possible return of which M. Velpeau would not commit himself. A microscopic examination of that tumor by MM. Robin and Verneuil, gave identical results, viz., absence of the cancerous element, the tumor being constituted especially of a hypertrophy of the secreting mammary cul-de-sacs, with atrophy of the secreting conductors.

I conclude from this, said M. Robert, that art is deficient; but I do not accuse the means which in this particular case, appears to me, to have surmounted obstacles that had arrested even M. Velpeau himself.

M. Robert complained that the discussion had been commenced without defining the subject of it: What is cancer? No one has defined that disease. The greatest disorder reigns in the nomenclature of solid tumors. * * * * The persevering employment of our ordinary methods, and long continued labors consecrated to the study of tumors, have produced certain very useful documents; but at the same time very insignificant results. It is to them that we owe that sad richness of empty words and comparisons, often very coarse, which to-day encumber pathology. What do all these words teach you? colloid cancer, tuberous, woody, napiform, chondroid, pustulous, encephaloid, schirrhus, lardaceous, medullary fungous, hematodes? * * * * Chancrous, eating, perforating ulcer, noli me tangere, etc.? What signification is there for you in the hardness or softness, or even the famous creak of the scalpel so peremptory in the past?

As to the cancer juice, as to the pulp, this sign is wanting sometimes, and is found when there is no cancer.

The microscope proposes for cancer a clear and precise definition. There is but one cancer, and that is the one which contains the cancerous element.

There are other morbid tissues which may be confounded with cancer, and which, sometimes, may cause death. These tissues result from an exaggerated production of one or several elementary substances, which normally enter into the constitution of our bodies. Finally, aided by the microscope, anatomists had better studied the intimate nature of tumors, and having learned their development and growth at the bedside, they have been able to discover differences between them and have proposed a revision of that part of nosology.

M. Robert does not hesitate to accept the definition of the microscopists with regard to the specificness of the cancer cell or germ, and he demands why M. Velpeau refuses to accept this definition. The development of cancer is also peculiar; its tendency to relapse also very marked; cachexy is frequently the result.

With regard to fibro-plastic tumors, M. Robert thinks that they should not be classed among benign tumors; but he denies that they should be confounded with cancer; their progress is slow, they rarely return, and even a cure may be effected after several relapses.

Coming to epithelial tumors, M. Robert says, that the comparison made by M. Velpeau between these tumors and corns and warts, is more specious than true. In effect if the element is the same, the structure is altogether different. The return of cancroid tumors is more rare than cancer; the infiltration of the neighboring ganglia is also very infrequent.

To resume: the microscope has been more useful in surgical diagnosis than M. Velpeau is willing to concede; and since the microscope has revealed to us the intimate structure of tumors by glandular hypertrophy, the diagnosis of these tumors, their progress and evolution, have been better studied: having been applied for only a few years to pathological anatomy, the microscope has already produced happy results. The young school, as M. Velpeau calls it, is then on the right track. It is necessary, then, for it to persevere, and resolutely go ahead, every day will have its progress.

During the same sitting, M. Barth presented a pathological specimen (cancer of the testicle,) and asked if the results given by the microscope and clinical experience, coincided; he pointed to the dangers which would result from an exclusive tendency to pay attention only to certain microscopic elements, to determine morbid productions.

M. Velpeau presented, also, several tumors whose character could be easily determined by the naked eye: according to his judgment, one was schirrhus, the other also cancer; there was no doubt with regard to them.

M. Gerdy protested against the expression of certitude employed by M. Velpeau; such certainty as M. Velpeau believes in cannot here be admitted.

*Sitting of October* 31*st*, 1854.

M. Malgaigne proposes to examine, at this sitting, three distinct questions:

What is the utility of the microscope in science?

What is the utility of the microscope in practice?

What is the degree of curability of cancer?

M. Malgaigne first examines the state of science before the application of the microscope to the study of tumors. Three schools have brought forth their doctrines since the commencement of this century. The first can be represented by Scarpa and Boyer; the second, by Bayle and Laennec; the third, or English school, by A. Cooper and Abernethy. Boyer guiding himself alone by the anatomical character of tumors, distinguished only the hard schirrhus, which return but rarely; from the soft schirrhus, which nearly always return. Scarpa was no more advanced. Bayle and Laennec admired the encephaloid, the schirrhus, the colloid and the melanic. Abernethy and A. Cooper delighted, the first in benign sarcoma, which comprehended adipose sarcoma, cystic sarcoma, etc., and malignant sarcoma, divided into tuberculous, schirrhus, medullary, etc.; and the last in benign tumors, which he called adipous, scrofulous and irritable, and malignant tumors divided into schirrhus, soft cancer, and fungoid cancer.

In 1844, M. Cruveilhier excited a discussion in the Academy, upon cancer, which he said should not be confounded with certain fibrous tumors of a benign nature. This distinction was not then admitted without objection. M. Velpeau was the first who had called to his aid the microscope, and he would never repent it; to the microscope belongs the honor of having entirely changed the face of science.

M. Malgaigne denies the proposition of Bichat, when two diseases differ in their symptoms, their march and their termination, they differ, also, in their location and the lesions which accompany them, and reciprocally when the anatomical lesions are different, when the structure of two pathological products is not the same, the functional disorder, their evolution constitute, also, affections of a distinct nature. This law is false. It has led M. Lebert to affirm that fibro-plastic and cancroid tumors acted in a very different manner from cancer. Experience showed him that he was wrong. M. Lebert, said M. Malgaigne, would have been perfectly right had he confined himself to anatomical distinctions and classifications. But as soon as M. Lebert left the anatomical to enter upon the practical, he run against impossibilities, and clinical facts immediately ruined his doctrines. With regard to benign tumors the microscope has not given us much light. Passing to the question of the curability of cancer, M. Malgaigne says that if he believes the cure of cancer very rare, he does not absolutely deny it.

During the same sitting, M. Larrey spoke in favor of the microscope. We cannot give a better idea of the sense of his remarks, conceived in a spirit of conciliation and liberality extremely laudable, than in transcribing a few of the last sentences: Gentlemen, I will say that the Academy is not yet in a condition to decide for or against the application of the microscope in the diagnosis and prognosis of tumors. I would wish, for my part, and I submit this proposition to the judgment of the Academy, I would wish that the cause of the microscopists was defended by them, or one of them, in behalf of his fellows, so that his arguments printed in our bulletins at the end of this discussion, would show to the public the community of our efforts to throw light upon this dark question of cancer, and leave us a hope, and not an illusion of its curability.

*In the same Sitting, Nov. 7th,*

M. Velpeau again took the floor. He commenced by calling to mind the two propositions which he sustained at the commencement of this discussion: first, the possibility to sometimes cure cancer by an operation; and in the second place, to diagnose cancer without the application of the microscope. In 1854, here in this Academy, he had been the first to speak of the cancer cell. "Be persuaded," said M. Velpeau, "that it has not entered into my mind to despise and condemn any means of investigation. I accept all, and make use of every method, and when they tell me something which does not accord with what I have seen, I make a note of it; but when what they announce is contrary to what experience has taught me, I reject it, at the same time of course permitting others to accept if they choose."

M. Velpeau does not admit that there is a cell characteristic of cancer, and there are microscopists who think as he does: MM. Mandl, Virchow, Bennett and Paget. * * * Epithelial cancer has no cells. * * * Laying argument aside, M. Velpeau invokes the results of his long practice. Since 1824 he has not ceased to make investigations on this question of cancer. * * * M. Velpeau has seen the cell in many cases, but there are cases where it is wanting, and it has not been found in many cases which for him and other surgeons were manifestly cancers. After such results he could not believe in the specificness of the cell. Besides the microscope has found the cell in tumors, which were certainly not cancers. M. Velpeau cited, among other examples of cancer without the cell, the case of a poor woman operated on by him a year ago, who is now dying, suffocated by a cuirasse of tumors which have sprung up, covering the whole chest. There the microscope did not find the cell and had given a favorable prognosis.

M. Velpeau mentioned the case of a man who had been operated on for a cancroid of the nose; it returned, and death followed. But the microscopic examinrtion, before the operation, did not discover the cell; afterward the cell was found in great abundance. A woman presented herself with a tumor of the left breast—M. Velpeau operated—the tumor contained no cells; but the disease returned, not only in the left, but in the right breast also, and the autopsy showed similar tumors in the lungs; but these tumors examined by the same microscopists, were declared to contain the cancer cell in great numbers. For M. Velpeau, the two diseases coming so close together are one and the same lesion. As for a transformation the microscopists were the first to reject it. Until something more is gained, M. Velpeau maintains that there are cancers without the cell, and benign tumors which contain it. The microscopists demand what is a malignant tumor, and they wish that we should define cancer. There are many things that we know better than we can define; but for the want of a better definition, I would be perfectly content with one like this: a tumor, a plaque, a fungosity, having the character when once established, to increase, to invade and cause to disappear the neighboring tissues: in one word, to invade the organism until death ensues. M. Velpeau calls malignant tumors, those which when once established in the organism, develope themselves and destroy the neighboring parts until they entirely disappear, or cause the death of the individual. There is no other tumor but cancer, which acts in this manner, and this term (malignant) serves to distinguish cancer from all other lesions.

M. Velpeau then showed that epithelial tumors reproduced themselves in the same manner as cancers; as to the frequency of their return, if it is less, this is not sufficient to strike out these tumors from the class of cancers.

Fibro-plastic tumors, no more than the preceding, are exempt from relapse, and they reäppear sometimes far from the point from which they had been removed. (M. Velpeau cited several facts in support of this opinion.)

After having given the case of a lady operated on by him for an enormous ulcerated cancerous tumor, which did not return, M. Velpeau came to the question of the curability of cancer. * * *

* * * "When I have given an affirmative diagnosis," said he, "I can guarantee its validity. Besides, the nature of these tumors cannot be learned (microscopically) until after the death of the patient, or after their removal; but in practice a *post-mortem* diagnosis is of no utility, and a diagnosis after an operation is of no use to guide the conduct of the surgeon." * * *

M. Velpeau produced statististics from which it appeared that the microscope had not disputed his diagnosis. Of 120 tumors for this year alone, he had found 28 adenoid, others hypertrophies. * * * M. Velpeau complains that the microscopists do not always agree upon the nature of tumors, and when some do not find the cell, others will discover it. * *

The facts upon which M. Velpeau sustains himself in affirming the curability of cancer, are numerous, and a few go back fifteen and twenty years without a return of the disease. There are cancers which, assuming a special form, are worse than others, and renew themselves with almost absolute certainty. For kinds so malignant and whose return is almost the infallible law, M. Velpeau does not operate, because the operation never cures; but it is by no means the same in all cancers.

M. Velpeau believes that cancer is a disease primitively local, the cancerous diathesis and infection coming on later.

M. Velpeau terminates in requesting physicians not to repudiate the examination of the patients for the examination of the tumors.

*Sitting of Nov. 21st,*

M. Amussat pronounced a discourse, the conclusion of which we transcribe:

1st. To avoid believing that cancer is an incurable affection, for that unhappy idea tends to diminish the zeal of our observers and surgeons who labor to push forward the progress of science; I believe to the contrary, that it is necessary to stimulate and encourage them by every possible meaus, and I have indicated those which appear to me the most proper to attain that end.

2d. In the present condition of science and practice, as soon as a patient is attacked with a cancerous affection, it is necessary, after having carefully examined him, to seek from him with discretion, but at the same time with persistency, all information with regard to the diseases which have been most frequent in his family; and if in this examination any hereditary taint should be manifest, it is necessary to act immediately with all the most efficacious means at our disposal.

3d. As soon as an operation is decided upon, whether by the bistoury or with caustics, it is necessary to go beyond, rather than stop this side of our aim, that is to say, to entirely destroy the cancer, and not to stop until the healthy parts are reached. Very often timidity in operations has been the only cause of relapse.

4th. When it is thought preferable to employ caustics, the most powerful should be used; avoid caustics which act slowly and superficially. I am not in favor of the red-hot iron, which is so frightful, and, in certain cases, dangerous.

5th. It is necessary to assure yourself, by a minute examination many times repeated after the apparent cure, that there is no trace of the cancerous affection left.

6th. Finally, until the true nature of cancer is understood, we should advise after operation, depuration, or a seton, and proper hygiene.

M. Cloquet, after having sought to distinguish tumors after certain characters, such as their being hereditary, liable to relapse, and incurable by aid of nature alone, said that the microscopists were only a continuation of the anatomical school. Lately, said he, they have applied the microscope in pathological anatomy; it is a step forward in the same direction, but there is nothing there, upon which to found a school or a new doctrine.

M. Cloquet thinks that there are cancers with cancer cells, so called; cancers with epithelial cells; and cancers with fibro-plastic cells.

M. Cloquet recognizes that there are differences in the march of cancer, and that these differences accord with the distinctions established by the microscope. He believes in the curability of cancer. * * * He terminates in begging the anatomists, the cliniciens, and microscopists, to unite their efforts to a common end.

M. Delafond, after having established that cancerous diseases are very frequent among herbivorous and carnivorous animals, discussed the question of the cancer cell. For him the cancer cell is not a specific element; it is similar in every character to all other cells making up the organization. This cell is regular and round at its birth; it becomes deformed when compressed; it is sometimes penetrated by pigmentary matter, * * * by fat molecules. After explaining at length the formation of vegetable existence, M. Delafond showed, by plates, the cell presiding at the birth of tissues; he thinks the method of sustaining vegetables is the same as that presiding in the sustenance of healthy animal organism. The cell is everywhere the same. What causes the nature of tissues to vary, is the different materials with which it (the cell) fills itself. * * * The cancer cell takes its growth in the midst of a liquid, which is the proper element of cancer. * * * * Once formed this cell will assume different forms, adapting itself to the conditions in which it finds itself. If compressed by a fibrous tissue very resistant, they unravel themselves to form veritable fibrilles, exactly as this takes place in normal tissues. * * * * * * M. Delafond, in support of his assertions, produced numerous plates where the different forms of the cell were visible.

He concludes that with regard to microscopic anatomy, there is no difference between encephaloid cancer the best characterized, and fibro-plastic tissue.

*Sitting of Nov. 28th.*

M. Delafond again took the floor, and brought new arguments to the support of the doctrine that he had advanced at the last sitting. He thus resumed his observations: "There exists, in animals as well as in vegetables, a cell which is active in the development of both; there exists besides the normal cell, another cell which may be called a pathological cell, of which the cancer cell is only a modification; this cell may exist not only in cancer, but in the fibro-plastic tissue in animals; this cell offers but little difference, whether it be examined in schirrhous, encephaloid, or fibro-plastic tissues, with regard to its form, structure, or dimensions, or the modifications impressed upon it by any of the common reäctives. I say, then, that in practice, the microscopic proof of the cancer cell is not sufficient to affirm whether the tumor be cancer or not; but that in diagnosis, the cell is a character which never should be neglected, because in most true cancers it is found, and its presence adds one more presumption to those which have existed from a clinical and anatomical examination of the tumor." * * *

*Sitting of Dec. 5th.*

M. Bouillaud took the floor in favor of the anatomical school. His remarks are difficult to analyze; to substitute the dryness of analysis for elegance and brilliancy, destroys the charm. The speaker raised his views to a grand elevation, and his style was upon a level with the subject that he treated. Placing himself on general pathology, M. Bouillaud lent the support of his talents and warm convictions, to the law of Bichat, attacked by M. Malgaigne.

What is true and incontestible in the law of Bichat, said M. Bouillaud, is, that it is in perfect accord with what we observe every day, in those diseases that I call common, which in truth are almost the exclusive object of our observations and practice; I say, gentlemen, that this law is true in all cases; I say that every day the phenomena observed on our patients, reveal to us the condition of their organs; I say that anatomical lesions make known to us the functional disorders which they had produced; and I do not know, in truth who would contest it; for if that were not so, there could be no more instruction, there would be nothing more to do but to close the schools.

Justifying the anatomical school from the reproaches heaped upon it, M. Bouillard affirmed that no one had ever said that the anatomical lesions would give the key to all of the problems of pathology. Passing in review the men who had been the founders of the Paris school, M. Bouillaud cited Morgagni, Haller, Corvisart, Pind, Bichat, Laennec, Broussais. A pure organic school never existed. This anatomical school which has been alluded to, does not exist; the school of Paris is organico-vitalist.

The anatomical diagnosis is useful for its precision; but it is not complete until a physiological diagnosis is joined to it.

Coming to the question of cancer, M. Bouillaud says that if the cell is so specific as the microscopists assert, it must be recognized that it is of immense value in the diagnosis of cancerous diseases. * * *

M. Bouillaud reproaches M. Velpeau with having been the decided adversary of the microscopists, no matter what he says to the contrary; and M. Delafond with having obscured the question by developing his ideas on the proteic cell.

With regard to the curability of cancer, M. Bouillaud will not deny its possibility, but makes a distinction between local cancer and the cancerous diathesis. * * *

M. Bouillaud demands, in conclusion, that a committee be appointed by the Academy to examine, in company with the microscopists, certain tumors selected for the purpose.

*Sitting of January 2d.*

M. Robert replied principally to the arguments of M. Delafond; to the designs produced by that speaker to prove the unity of the cell, and brought forward other designs representing very different forms of cancer; also the fibro-plastic and epithelial elements. * * *

M. Delafond again defended his doctrine of unity of the cell * * *

sustaining himself by respectable authorities, such as Purkinje, Turpin, Valentin, Schluttz, Wagner, Bischoff, Martin, Barry, Vogel, Müller, Henle, etc., he proved that this doctrine was far from being abandoned. The speaker cited in favor of his opinion, passages borrowed from the writings of several modern authors, Vogel, Virchow, Bennett, * * * and concluded that M. Robert had yet shown nothing to the assembly proving any difference between normal cells and cancer cells. M. Delafond entered into practical details with regard to the size of the germs and the cells in animals and in man. * * * He terminated by conclusions, the last of which is a perfect resumé of his opinions:

". These facts show that the blastema, the morbid principle of cancer, resides in an amorphous principle, unseizable by our present means of investigation, and independent of these fibro-plastic and epithelial cells, and of the cell improperly called cancerous."

*Sitting of January* 9.

M. Leblanc replied to M. Delafond, and spoke principally upon points interesting to the veterinary surgeon, viz., the relative frequency of cancer in herbiverous and carniverous animals. * * *

*Sitting of January* 16.

M. Velpeau insists upon the point that tumors may not present the same composition at every period of their development, that is to say, at the commencement they may nôt contain the cell — but later it may be found. On the other hand, a tumor may return and the cell may not be found, when it was found previous to the operation. M. Velpeau brings up the case of a woman, who had on the left side of the chest a tumor of doubtful character, and who afterward presented a tumor of the other side of the chest, whose character was evident; in the doubtful tumor the microscope discovered the cell, while in that one which was unquestionably cancer no cell was found. * * M. Velpeau proves by facts that epithelial and fibro-plastic tumors, return like cancer, and are capable of multiplying themselves and appearing in different parts of the body. With regard to the term malignant, M. Velpeau cannot explain why some persons feign to be unable to comprehend this word. * * * It is the essence of the tumor which constitutes its malignity; a cancer is malignant, whatever be its size, age, or position, * * * but there are degrees of malignity among cancers. There is but the cancer (whether it has the epithelial or fibro-plastic cell) which presents constantly this character of malignity, whose inevitable consequence is death.

M. Velpeau wishing to show how much confusion microscopy has introduced in the nomenclature of tumors, thus expresses himself:

"I remove in the same week a keloide, a hard, horny, indolent tumor, which never ulcerates and has never caused death. What is this? It is fibro-plastic, replies the microscope. I then remove an engorged lymphatic ganglion, a tumor essentially benign, without doubt. What is this? Fibro-plastic. I removed next a soft tumor of gummy consistence, from the calf of the leg; it was a mass resembling one of those fibrinous concretions found in the heart of some individuals after death. What is this? Fibro-plastic. I remove some of those fungosities found around articulations. Still fibro-plastic. Finally, I ask of them what that hard, woody substance is forming the base of a venereal chancre? Still fibro-plastic. But see, gentlemen, how all of these resemble one another: among these tumors are hard and soft; some transient and some persistent; there are some which never reproduce themselves and some which return with the greatest obstinacy. But it is the microscope that has said it; all these form a group excessively natural. Before we were in a chaos of confusion: see how we can profit from this new light."!!

"Let us continue: here upon the lip is a little tumor which has just commenced to grow; nevertheless that little tumor will inevitably produce death. What is it? It is epithelial. Here is a corn on the toe; what is it? Epithelial. And this fungosity on the mucous membrane of the anus; what is it? Still epithelial. And this little concretion raising the conjunctiva? Epithelial. And upon this tongue here is a tumor which goes on increasing until death ensues, if it is not removed in time, and even then will almost certainly return and cause death; this is still an epithelial tumor. Aud in this uterus there is a tumor soft as cheese, and in other organs tumors hard or horny. What is all this? Epithelium, always epithelium. There is light."!! * * *

M. Velpeau is, and has always been, an anatomist, but he takes the clinic as a point of departure, and seeks to diagnose disease during life.

* * * After having reproduced the arguments which had already been exposed in the course of the discussion, M. Velpeau terminates with kind words for the microscopists, to whom, however, he makes no concessions.

After stating where these discussions are published in full, the writer thus resumes:

And now can any one state the opinion of the Academy with regard to cancer and the microscope? Does the Academy believe that cancer is curable? Does it believe the microscope useful in the progress of pathological anatomy, and aids in the diagnosis of tumors? We will not attempt to be the interpreter of the opinion of the Academy.

It is incontestible that no question has so excited the medical public, that never had so much talent and feeling been expended in a scientific discusston. Here are the results of all these efforts and this passionate strife:

Matters remain as before the discussion; but the question has been laid down, science arrested at a given moment, and it has been possible to verify the state of our knowledge on this subject in the year 1855. It is now impossible to go back; it is necessary to choose between the clinic alone and the clinic aided by anatomy.

For us, who believe in the progress of anatomy in general, both normal and pathological, and who do not believe that the cliniciens can dispense with the anatomists, we will continue to have faith in the future of the microscope applied to medicine, and we will not accept the anathema with which this precious means of knowledge has been struck.

All the reproaches addressed to the anatomists, only signify one thing: that they do not know anatomy well enough. They will study it better, and the cliniciens will be benefitted in spite of themselves, from the labors of the anatomists. But is it not permitted to think, that the differences at present manifest between the members of the medical family, who should agree, will disappear when it will be proved that there are anatomical facts, and clinical facts, and that anatomy cannot bend itself to the exigencies of medical theories.

Perhaps this misunderstanding would have been avoided if the base on which the discussion should rest, had been established before commencing it. Before agitating the question of the curability of cancer, it would have been better to define cancer; but if it is defined clinically, the discussion would be between the cliniciens: and if anatomically, between the anatomists. Unhappily confusion reigned during the whole course of the discussion.

The modern microscopists acquired their education at the schools of clinicens, and conducted by the hand, they have followed the wanderings of their teachers; they have studied with preconceived notions; they learned the word cancer and have sought for cancer. * * * They have had the hope or illusion of finding this ideal type; they took their cue in consequence and promised what was demanded of them. Now they are set free from the tutelage which guided their first steps; they exist of themselves; they have opinions of their own, which by some are repudiated. Why then call on them for aid? They can only tell what they see; anatomical facts are imposing; they cannot be made use of, but they cannot be denied. If microscopic anatomy is an evil it is an evil with which it is necessary to accustom ourselves to live henceforth. Young men who are not responsible for the past, will devote themselves to microscopic anatomy, and by this means will learn things which it was not given to their ancestors to understand.

